# Comparative safety for cardiovascular outcomes of DPP-4 inhibitors versus glimepiride in patients with type 2 diabetes: A retrospective cohort study

**DOI:** 10.1097/MD.0000000000007213

**Published:** 2017-06-23

**Authors:** Hyouk-Jun Chin, Jin Hyun Nam, Eui-Kyung Lee, Ju-Young Shin

**Affiliations:** School of Pharmacy, Sungkyunkwan University, Suwon, South Korea.

**Keywords:** cardiovascular outcomes, DPP-4 inhibitors, glimepiride, type 2 diabetes treatment

## Abstract

Concerns about the cardiovascular safety of dipeptidyl peptidase-4 (DPP-4) inhibitors persist. This study sought to determine whether there is a differential risk of hospitalization for cardiovascular diseases (CVDs) between DPP-4 inhibitors and glimepiride.

We conducted this retrospective cohort study by using the Korean National Health Insurance Service database from December 1, 2008, to December 31, 2013. The study subjects were new users of DPP-4 inhibitors or glimepiride for type 2 diabetes. Outcome was defined as hospitalization for CVDs, including angina pectoris, myocardial infarction, transient cerebral ischemic attack, heart failure, or cerebrovascular disease or any procedure involving coronary artery bypass grafting or percutaneous coronary intervention. We used a Cox proportional hazard model to estimate the adjusted hazard ratios (aHRs) and their 95% confidence intervals (CIs), to assess the risk of CVDs associated with the use of DPP-4 inhibitors compared with glimepiride.

The cohort consisted of 1,045,975 patients, with 6504 in the DPP-4 inhibitors group and 13,447 in the glimepiride group. No significant increased risk of total CVDs was found (aHR, 0.87; 95% CI, 0.75–1.01) in the DPP-4 inhibitors versus glimepiride group. A decreased risk of hospitalization for CVDs was found among patients with a history of visit for CVDs (aHR, 0.73; 95% CI, 0.56–0.97) or with >2.5 years’ duration of type 2 diabetes (aHR, 0.77; 95% CI, 0.66–0.91) in the DPP-4 inhibitors versus glimepiride group.

DPP-4 inhibitors did not increase cardiovascular risk compared with glimepiride regardless of CVD history and diabetes duration.

## 1 Introduction

Dipeptidyl-peptidase-4 (DPP-4) inhibitors, which are relatively new antidiabetic drugs that have been available since 2006, are prescribed clinically worldwide for patients with type 2 diabetes mellitus (T2DM) because of their unique insulinotropic action, low risk for hypoglycemia, and low risk for associated weight gain.^[1]^ However, there have been concerns about the effect of DPP-4 inhibitors on cardiovascular diseases (CVDs) because of repeated reports about DPP-4 inhibitors positively or negatively influencing the cardiovascular system.^[2,3]^

Several prospective clinical trials for evaluating the effect of DPP-4 inhibitors on CVDs have been published. In the SAVOR-TIMI 53 (saxagliptin assessment of vascular outcomes recorded in patients with diabetes mellitus—thrombolysis in myocardial infarction 53) trial, patients taking saxagliptin were more likely to be hospitalized for heart failure (HF) than those in the placebo group (hazard ratio [HR], 1.27; 95% confidence interval [CI], 1.07–1.51).^[4,5]^ In the EXAMINE (examination of cardiovascular outcomes with alogliptin versus standard of care) trial, patients taking alogliptin did not increase the risk of hospital admission for HF than those in the placebo group (HR, 1.07; 95% CI, 0.79–1.46). Alogliptin had no effect on composite events of cardiovascular death and hospital admissions for HF in the post hoc analysis (HR, 1.00; 95% CI, 0.82–1.21).^[6]^ Most recently, the TESCO (trial evaluating cardiovascular outcomes with sitagliptin) trial demonstrated that sitagliptin was not associated with a risk of hospitalization for HF.^[7]^ Observational studies were conducted using real-world data to evaluate cardiovascular outcome risks; however, the results were conflicting.^[8–16]^ A retrospective observational study in which a US insurance claims database was used to compare DPP-4 inhibitors and sulfonylureas showed no association between HF or other selected cardiovascular outcomes and DPP-4 inhibitors^[16]^; in a large retrospective cohort study, incretin-based drugs were not associated with an increased risk of hospitalization for HF.^[17]^ However, in 1 report of a population-based study, the use of sulfonylureas increased the risk of hospitalization for HF.^[11]^

Despite previous clinical trials and observational studies, it remains uncertain whether individual DPP-4 inhibitors have differential cardiovascular effects.^[5,18]^ Among the previous studies, most randomized controlled trials compared the relative risk of a specific DPP-4 inhibitor with placebo instead of an active comparator compound. Moreover, previous observational studies analyzed 1 or a few individual DPP-4 inhibitors^[9,10]^ and could not document a sufficient long-term follow-up time for evaluating the cardiovascular outcome.^[16]^ Cardiovascular risk may vary among DPP-4 inhibitors, and these drugs can be categorized according to their nonpeptidomimetic characteristics (referring to the noncovalent extracellular cross-talk with residues in the catalytic site of the DPP-4 substrate, resulting in a strong and immediate inhibition as opposed to peptidomimetics, which show lasting inhibitory activity).^[2]^

Therefore, the purpose of this observational cohort study was to evaluate the association between the use of DPP-4 inhibitors and the risk of CVDs compared with glimepiride, by using the Korean National Health Insurance Service (NHIS) database. We also sought to evaluate the differential risk of each DPP-4 inhibitor.

## 2 Materials and methods

### 2.1 Data sources

We collected patient data from the NHIS database, including approximately 1 million persons extracted randomly from almost the entire South Korean population, totaling 51 million people, by using national claims data from January 1, 2002, to December 31, 2013. In this database, various variables were included such as sex, socioeconomic status, medical care history (medical treatment and health examination), medical care institution, diagnosis code, surgery code, and drug prescription data (drug name, dosage, and date of prescription).

### 2.2 Study subjects

Each participant was ≥20 years of age, had at least 1 recorded diagnosis of T2DM (International Classification of Diseases [ICD]-10 codes E11–14), and were newly prescribed with at least 1 blood glucose lowering drug or insulin between December 1, 2008 and December 31, 2013, without having had any prescriptions during the preceding year. Index date was the first prescription date of a DPP-4 inhibitor or glimepiride. Patients with a prescription history of insulin or a GLP-1 receptor agonist, within a year before the index date, were also excluded to remove the effect of the same incretin-based therapy with a DPP-4 inhibitor and implications for the management of diabetes.^[19]^ Patients with a diagnosis of pancreatic cancer (ICD-10 code C25) were also excluded because most of these patients have glucose intolerance or diabetes.^[20,21]^

### 2.3 Exposure assessment

**Figure 1:**
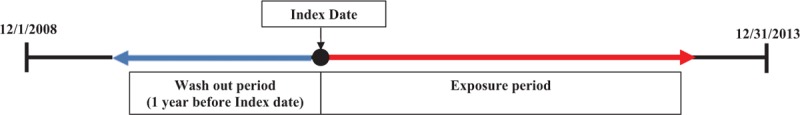
Description of study periods.

DPP-4 inhibitors included vildagliptin, saxagliptin, sulfonylureas, linagliptin, and gemigliptin. Exposure started on the date of the first medication of a DPP-4 inhibitor or glimepiride after the eligibility period. The index date was the first prescription date of the study drugs. Subjects were considered exposed until the end of the continuous exposure period (Fig. 1). A 15-day grace period between study drug periods was allowed before assuming that the medication was discontinued. Subjects who discontinued or switched index drugs were censored at the date of switching or discontinuation.

### 2.4 Study outcomes

The outcome of interest for this study was defined as any hospitalization or visit to an emergency department because of CVD with a primary diagnosis of angina pectoris (ICD-10 code I20), myocardial infarction (ICD-10 codes I21, I22, I23, I25.0, and I25.1), transient cerebral ischemic attack (ICD-10 code G45), heart failure (ICD-10 code I50), cerebrovascular disease (ICD-10 codes I60, I61, I62, I63, I64, I65, and I66), or any procedure involving coronary artery bypass grafts or percutaneous coronary intervention. The outcome date was determined as the earliest date when a given outcome was diagnosed in a patient.

### 2.5 Potential confounders

The potential confounders were sex, age at index date, duration of diabetes, diabetes-related complications, family history of diabetes mellitus, Charlson comorbidity score, comorbidities, and comedications. The duration of diabetes was defined as the duration between the date of the first diabetes diagnosis and the index date. All comorbidities and comedications were assessed during 1 year before the index date.

Diabetes-related complications included retinopathy, neuropathy, nephropathy, and peripheral vascular disease. Charlson comorbidity score was also estimated from the disease record by using previously validated algorithms.^[22,23]^ Comorbidities included hypertension, dyslipidemia, obesity, chronic obstructive pulmonary disease, and hypoglycemia. Finally, we assessed the use of blood glucose lowering drugs and comedications within 1 year before the index date. Blood glucose lowering drugs included metformin, sulfonylureas, thiazolidinediones, α-glucosidase inhibitors, and meglitinides. Other comedications included angiotensin-converting enzyme (ACE) inhibitors, angiotensin II receptor antagonists, β-adrenergic antagonists, calcium channel blockers, thiazide diuretics, other diuretics, nitrates, digoxin, aspirin, other antiplatelet drugs, warfarin, new oral anticoagulants, other anticoagulants, and statins.

### 2.6 Statistical analysis

We used descriptive statistics to compare the characteristics between the 2 groups. Values are described as mean and standard deviation or frequencies with proportions. We also estimated the incidence of each outcome in the DPP-4 inhibitors and glimepiride groups. We created Cox proportional hazards models to estimate crude and adjusted HRs (aHRs) and their 95% CIs for the risk of CVDs associated with the use of DPP-4 inhibitors compared with glimepiride. The adjusted model included sex, age, duration of diabetes, history of CVDs, treatment duration, use of other blood glucose lowering drugs, diabetes-related complications, Charlson comorbidity index, presence of chronic obstructive pulmonary disease, and comedications (ACE inhibitors, angiotensin II receptor antagonists, β-adrenergic antagonists, calcium channel blockers, thiazide diuretics, other diuretics, nitrates, digoxin, aspirin, other antiplatelet drugs, warfarin, new oral anticoagulants, other anticoagulants, and statins).

We conducted stratified analyses according to diabetes duration, history of CVDs, treatment duration, use of blood glucose lowering drugs, total number of blood glucose lowering drugs used, use of ACE inhibitors, use of β-adrenergic antagonists, and use of angiotensin II receptor antagonists. We also repeated our analysis according to the peptidomimetic characteristics of the DPP-4 inhibitors. Vildagliptin and saxagliptin were peptidomimetic DPP-4 inhibitors, whereas the others were nonpeptidomimetic DPP-4 inhibitors. Probability (*P*) values <.05 were considered significant. SAS 9.4 software (SAS Institute Inc., Cary, NC) was used for statistical analysis.

### 2.7 Ethical approval

This study protocol was approved by the Institutional Review Board of Sungkyunkwan University (no. SKKU-2016-09-013). Informed consent was waived by the board.

## 3 Results

### 3.1 Study population characteristics

**Figure 2:**
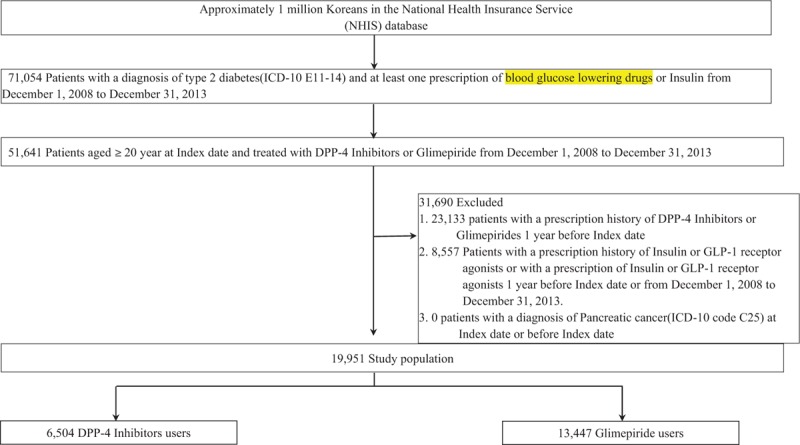
Selection of the study population.

In total, 19,951 patients were included in the cohort: 6504 in the DPP-4 inhibitors group (sitagliptin, 3541; vildagliptin, 1491; linagliptin, 1110; saxagliptin, 231; gemigliptin, 132) and 13,447 in the glimepiride group (Fig. 2). Patients with diabetes duration >2.5 years were more likely to be in the DPP-4 inhibitors group than in the glimepiride group (88.2% vs. 76.7%, *P* < .0001). The DPP-4 inhibitors group had more patients with a history of CVDs than the glimepiride group (18.1% vs. 12.7%, *P* < .0001). Patients in the DPP-4 inhibitors group had more diabetes-related complications and less couse of other blood glucose lowering drugs than glimepiride, excluding metformin and sulfonylureas (except glimepiride). The DPP-4 inhibitors group had a higher mean Charlson comorbidity score than the glimepiride group (Table 1).

**Table 1:**
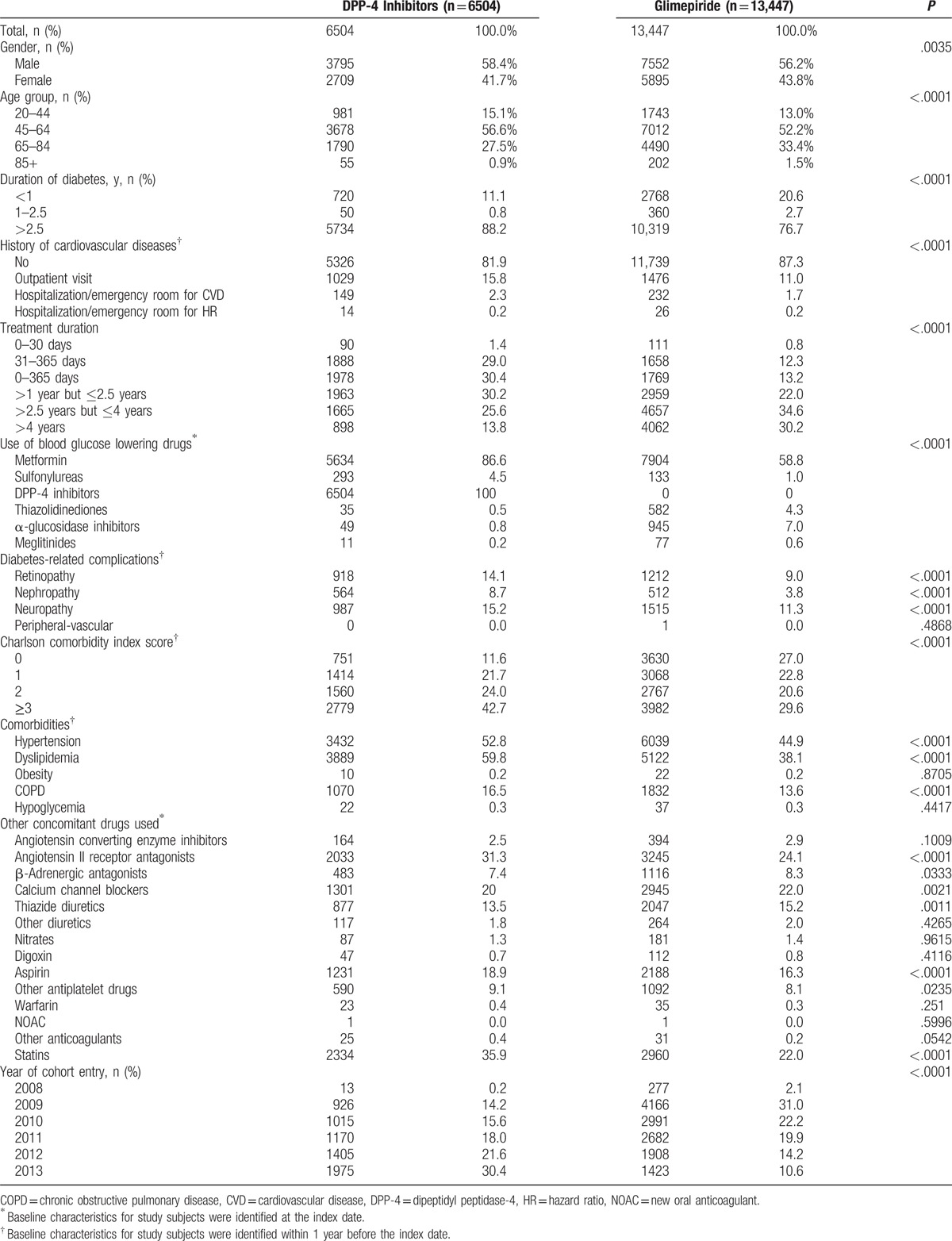
Baseline characteristics of the study population.

### 3.2 Incidence rate and HRs of study outcomes

The incidence per 1000 person-years for hospitalization caused by CVDs was 27.45 in the glimepiride group and 27.58 in the DPP-4 inhibitors group. No significant increased risk of total CVDs was found (aHR, 0.87; 95% CI, 0.75–1.01) in the DPP-4 inhibitors group with respect to glimepiride. Furthermore, no significantly increased risk of myocardial infarction plus angina pectoris (aHR, 0.93; 95% CI, 0.76–1.15) and cerebrovascular disease plus transient cerebral ischemic attack (aHR, 0.98; 95% CI, 0.81–1.20) was found. However, a decreased risk of hospitalization because of HF was also found (aHR, 0.58; 95% CI, 0.37–0.89) (Table 2).

**Table 2:**
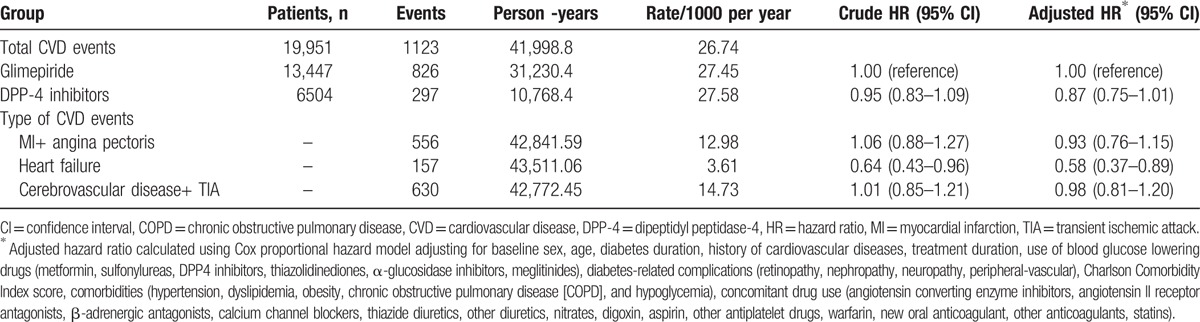
Hazard ratios of hospitalization for cardiovascular disease in patients treated with DPP-4 inhibitors versus glimepiride.

### 3.3 Stratified analysis

In the stratified analysis, the aHRs for DPP-4 inhibitors compared with glimepiride did not increase as diabetes duration and treatment duration increased. Patients with T2DM with histories of CVDs did not increase risk of hospitalization because of CVDs. In addition, there was no high risk of hospitalization because of CVDs among patients coprescribed with ACE inhibitors or angiotensin II receptor antagonists or β-adrenergic antagonists (Table 3).

**Table 3:**
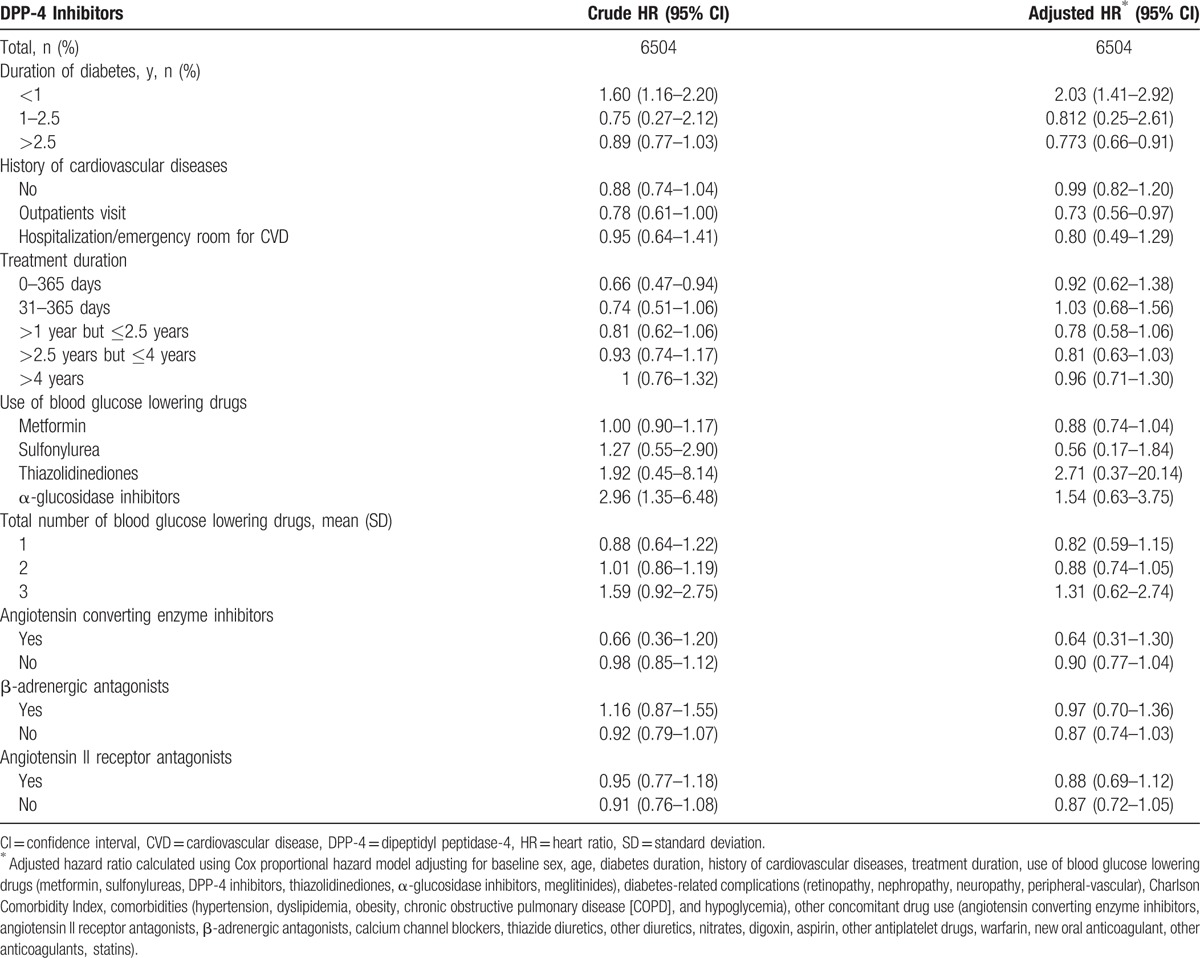
Hazard ratios for hospitalization by total cardiovascular disease in stratified analysis.

### 3.4 HRs in the DPP-4 inhibitors group

The aHRs of each of the 5 DPP-4 inhibitors are shown in Table 4. The aHRs of hospitalization for CVDs for peptidomimetic DPP-4 inhibitors and nonpeptidomimetic DPP-4 inhibitors compared with glimepiride were 0.82 (95% CI, 0.65–1.03) and 0.87 (95% CI, 0.74–1.03), respectively. Among the peptidomimetics, the aHRs for vildagliptin and saxagliptin were 0.77 (95% CI, 0.60–0.98) and 1.43 (95% CI, 0.78–2.63), respectively. It seems that saxagliptin is more likely to increase the risk of hospitalization for CVDs. Among the nonpeptidomimetics, the aHRs for sulfonylureas, linagliptin, and gemigliptin were 0.90 (95% CI, 0.76–1.07), 0.67 (95% CI, 0.41–1.08), and 0.60 (95% CI, 0.15–2.42), respectively.

**Table 4:**
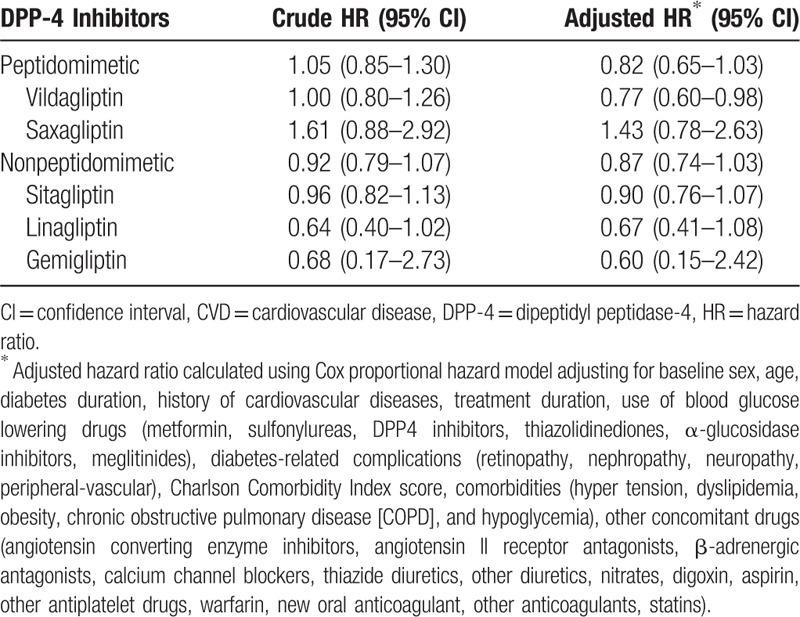
Hazard ratios of hospitalization for CVD by DPP-4 inhibitor group.

## 4 Discussion

In our retrospective cohort study, we found that DPP-4 inhibitors were not associated with increased risks of CVD-related events compared with glimepiride. DPP-4 inhibitors did not increase cardiovascular risk compared with glimepiride regardless of history of CVDs, diabetes duration, and treatment duration. It was previously documented that a few DPP-4 inhibitors increased the risk of hospitalization for HF. However, we found no risk of hospitalization for HF in the DPP-4 inhibitors group compared with the glimepiride group. This study provides evidence demonstrating the long-term beneficial effect of the use of DPP-4 inhibitors on CVD risk compared with glimepiride.

### 4.1 Comparison with previous studies

This study demonstrated that DPP-4 inhibitors did not increase the risk of hospitalization for CVDs compared with glimepiride (aHR, 0.87; 95% CI, 0.75–1.01). The results of the present study correspond well with those of previous studies showing that the CVD (including HF) hospitalization rate did not increase with the use of incretin-based drugs or DPP-4 inhibitors compared with the use of oral antidiabetic drugs in patients with diabetes.^[12,16,17,24]^

Patients with a previous outpatient visit because of CVDs are at a lower risk of hospitalization for CVD than patients taking glimepiride (aHR, 0.73; 95% CI, 0.56–0.97). This result is in agreement with the finding that DPP-4 inhibitors were not associated with a high risk of hospitalization for HF in patients with preexisting HF.^[25]^ Moreover, a systematic review revealed no statistically significant difference between studies on patients with and without baseline CVD.^[26]^

Interestingly, we found that the saxagliptin group seemed to have a tendency toward an increased risk of hospitalization for cardiovascular events compared with the glimepiride group (aHR, 1.43; 95% CI, 0.78–2.63). This finding is consistent with those of previous trials in which saxagliptin treatment significantly increased the risk of HF, especially among patients with a high cardiovascular risk, whereas no such increase was detected with other DPP-4 inhibitors except saxagliptin.^[18]^ Furthermore, vildagliptin, a peptidomimetic DPP-4 inhibitor similar to saxagliptin, demonstrated a decreased risk of hospitalization for CVDs (aHR, 0.77; 95% CI, 0.60–0.98). Therefore, we found that categorization of DPP-4 inhibitors into peptidomimetic or nonpeptidomimetic types from a pharmacokinetic point of view did not affect cardiovascular risk.

We also found several noteworthy results about an association between diabetes duration and DPP-4 inhibitors. In this study, the risk of hospitalization due to CVD for DPP-4 inhibitors was evaluated with diabetes durations of <1 year, 1 to 2.5 years, and >2.5 years. The result showed that DPP-4 inhibitors showed decreased cardiovascular risk with a long diabetes duration (>2.5 years), compared with glimepiride. Therefore, we found that for patients with short- and long-term T2DM, DPP-4 inhibitors could be used as antidiabetic agents to achieve optimal glycemic control.

To study the potential interaction between DPP-4 inhibitors and ACE inhibitors, we conducted a stratified analysis with ACE inhibitors, as it was previously documented that sulfonylureas interacted with high-dose enalapril and decreased blood pressure levels but increased heart rate as well as the levels of plasma norepinephrine and substance P, a member of the tachykinin neuropeptide family that increases heart rate and the vascular release of norepinephrine during couse of DPP-4 inhibitors and ACE inhibitors.^[27,28]^ The present study showed that DPP-4 inhibitors did not increase cardiovascular risk when used concomitantly with ACE inhibitors. We also found no effect of angiotensin II receptor antagonists or β-adrenergic antagonists with DPP-4 inhibitors on cardiovascular risk compared with glimepiride.

Contrary to the concerns about an increased cardiovascular risk with the use of DPP-4 inhibitors, which were triggered by randomized controlled trials with placebo control, our study found a protective effect on cardiovascular outcome, particularly against HF, with the use of the active comparator drug glimepiride. In addition to the glucose-lowering effect of DPP-4 inhibitors, there are several potential mechanisms underlying the potential beneficial cardiovascular effect of these drugs. First, the reported cardioprotective actions by DPP-4 inhibitors ranged from improving left ventricular ejection fraction to delaying the development of HF through the direct or indirect impact on cardiomyocytes, blood vessels, and blood pressure control.^[29–31]^ Second, previous studies reported that DPP-4 inhibitors might be capable of altering the mechanisms of molecules such as mitogen-activated protein kinases and nuclear factor kappa-B, improving endothelial function, decreasing inflammatory markers, and reducing ischemia/reperfusion injury in experimental models to decrease cardiovascular adverse events.^[2,32–34]^ This study can provide evidence supporting the above hypothesis about the advantageous action of DPP-4 inhibitors on CVDs.

This study has several strengths. First, we used the data of 19,951 patients with T2DM extracted from the NHIS database. Therefore, in this study, representative outcomes from almost all South Korean people can be identified in real-world clinical practice. Second, this study showed the HR for each DPP-4 inhibitor. In most previous observational studies,^[8,13,16,19,35,36]^ the HR of the DPP-4 inhibitor class was compared with that of other classes of antidiabetic agents. Therefore, in this study, the HRs were compared among 5 DPP-4 inhibitors, and the results can merit evaluating each DPP-4 inhibitor under the same analytical condition. Third, we used glimepiride as the active comparator drug in this study. Compared with most prescribed sulfonylureas, including glyburide, chlorpropamide, and glibenclamide, glimepiride may be considered safer in terms of cardiovascular risks because it does not block the myocardial protection afforded by ischemic preconditioning, produces smaller changes in ST elevation, and has less effect on coronary flow/resistance.^[37–39]^ Therefore, it is particularly important to compare DPP-4 inhibitors with glimepiride to reflect actual clinical practice. Henceforth, this study result is comparable with the CAROLINA study, an ongoing long-term actively controlled study of linagliptin and glimepiride in patients with T2DM.^[40]^ Fourth, in this study, about 5 years’ worth of NHIS data were used, with a mean follow-up period of 2.1 years for the 5 DPP-4 inhibitors, including the recently introduced DPP-4 inhibitor gemigliptin. This study had a rather long follow-up period compared with previous observational studies.

Our study also has some limitations. First, only patients whose condition was diagnosed at the hospital were considered in the cardiovascular outcomes. Therefore, cases with CVDs that required hospital admission were not analyzed. This might have underestimated the cardiovascular incidences in both groups. Second, some of the stratified analyses had insufficient statistical power for assessing cardiovascular risks because of the small sample size. Third, despite the strong adjustment for potential confounders, unconsidered confounders persist. This is a general limitation of retrospective cohort studies.

In conclusion, our data suggest that DPP-4 inhibitors do not significantly increase the risk of hospitalization for HF compared with glimepiride. Thus, the DPP-4 inhibitor class can be an alternative to glimepiride for treating T2DM because DPP-4 inhibitors do not seem to affect the risk of CVDs. The cardiovascular risk of patients with diabetes taking DPP-4 inhibitors is not associated with diabetes duration, history of CVDs, or duration of DPP-4 inhibitor treatment.

## Acknowledgment

This study used National Health Insurance Service (NHIS)—National Sample Cohort data (REQ0000006111) made available by the NHIS.
